# Structural and Functional Connectivity Changes in the Brain Associated with Shyness but Not with Social Anxiety

**DOI:** 10.1371/journal.pone.0063151

**Published:** 2013-05-10

**Authors:** Xun Yang, Keith Maurice Kendrick, Qizhu Wu, Taolin Chen, Sunima Lama, Bochao Cheng, Shiguang Li, Xiaoqi Huang, Qiyong Gong

**Affiliations:** 1 Huaxi MR Research Center, Department of Radiology, West China Hospital of Sichuan University, Chengdu, PR China; 2 Department of Psychiatry, State Key Lab of Biotherapy, West China Hospital of Sichuan University, Chengdu, PR China; 3 Key Laboratory for Neuroinformation, School of Life Science and Technology, University of Electronic Science and Technology of China, Chengdu, PR China; National Yang-Ming University, Taiwan

## Abstract

Shyness and social anxiety are correlated to some extent and both are associated with hyper-responsivity to social stimuli in the frontal cortex and limbic system. However to date no studies have investigated whether common structural and functional connectivity differences in the brain may contribute to these traits. We addressed this issue in a cohort of 61 healthy adult subjects. Subjects were first assessed for their levels of shyness (Cheek and Buss Shyness scale) and social anxiety (Liebowitz Social Anxiety scale) and trait anxiety. They were then given MRI scans and voxel-based morphometry and seed-based, resting-state functional connectivity analysis investigated correlations with shyness and anxiety scores. Shyness scores were positively correlated with gray matter density in the cerebellum, bilateral superior temporal gyri and parahippocampal gyri and right insula. Functional connectivity correlations with shyness were found between the superior temporal gyrus, parahippocampal gyrus and the frontal gyri, between the insula and precentral gyrus and inferior parietal lobule, and between the cerebellum and precuneus. Additional correlations were found for amygdala connectivity with the medial frontal gyrus, superior frontal gyrus and inferior parietal lobule, despite the absence of any structural correlation. By contrast no structural or functional connectivity measures correlated with social or trait anxiety. Our findings show that shyness is specifically associated with structural and functional connectivity changes in cortical and limbic regions involved with processing social stimuli. These associations are not found with social or trait anxiety in healthy subjects despite some behavioral correlations with shyness.

## Introduction

Shyness is a core dimension of temperament or personality trait that describes hesitation or discomfort in response to social situations, particularly novel ones [1]. It is an ubiquitous trait that over 90% of the population is reported to have experienced at some point in their lives [2]. For many individuals shyness occurs only during childhood, however 10–25% of the population have an enduring shyness temperament [3], [4]. Findings from a number of longitudinal studies have shown that shyness is one of the stable and heritable constructs which can predict important life outcomes in adulthood, such as interpersonal relations, occupational attainment and psychopathology [5]. However, there is still debate as to whether shyness as a personality trait is distinct from social anxiety which, while present in healthy populations can also become a clinical disorder under DSM-IV criteria. Indeed, a number of studies have reported significant correlations between shyness and social anxiety scores in healthy subjects [6], although only around 40% of subjects with the highest shyness levels also have social anxiety disorder [7].

To date, an emerging body of research studies has identified biological influences on shyness. For example, a significant association has been observed between the short allele of polymorphism in the serotonin transporter (5-HTTLPR) and shyness [3]. However similar association has also been reported with anxiety [8], [9]. Moreover, such biological influences interact with social environment. In a study of nonhuman primates, the interaction of maternal care giving and the 5-HTT promoter polymorphism predicted fearfulness [10]. Preliminary studies in humans also implicate 5-HTT in gene-environment interaction [11]. Given that genetic and environmental factors are linked with differences in brain structure [12], [13], we therefore hypothesized that interactions between them might also be reflected in the structural changes in the brains of shy and/or anxious individuals. Structural changes in the brain have already been reported associated with personality and temperament types [14]–[16] and can also occur quite rapidly, for example, when learning to master a new skill [17], [18]. It is therefore possible that genetic and environmental influences on temperamental shyness may also result in similar structural changes and these may or may not be distinct from those associated with anxiety.

On a more macroscopic level, personality traits or temperament represent tendencies to manifest particular patterns of cognitive, emotional and motivational behaviors in response to a variety of eliciting stimuli [19]. These tendencies are posited to arise from differences in the functioning of relevant brain systems controlling processing of social and emotional information. A number of fMRI studies have revealed that shyness is associated with hyper-responsivity to social stimuli in the amygdala [20]–[22] and frontal cortex [23]. A recent study by Kagan's group found greater amygdalar activation in response to novel faces, using fMRI in young adults who were classified as shy versus non-shy as children [22]. However, once again a number of studies have also linked social anxiety with hyper-responsivity in frontal cortex and amygdala in response to negative social stimuli [24].

Electrophysiological studies have shown that the pattern of resting frontal electroencephalogram (EEG) is associated with individual differences in shyness [1], [25], [26]. These EEG studies have found that the N2, N400 and other frontal negative ERP (Event-related potentials) amplitudes are enhanced during negative mood induction conditions, and among individuals reporting heightened levels of trait anxiety and internalizing symptoms. Furthermore, the medial prefrontal cortex (MPFC) plays a pivotal role in the modulation and inhibition of excessive limbic activity [27], [28]. Thus overall there is strong support for the hypothesis that shyness and/or social anxiety might be associated with altered limbic-cortical connectivity during emotional and cognitive processing.

However, despite this recent progress in identifying altered responses in the brain of shy individuals in task-dependent fMRI studies we do not know if there are any underlying structural or resting state functional connectivity differences. Assessments of brain function at resting state have become an increasingly useful way of investigating brain-wide alterations in functional cerebral networks as distinct from task-related differences involving only dynamic changes in a small number of regions [29]–[31]. Patterns of resting state functional connectivity are stable and consistent across time [32], [33] and across studies [34] and show differences associated with personality, such as impulsivity [35], risk-taking [36], and five-factor personality traits [37]. To date, however, no study has investigated potential resting-state functional differences in the brain associated with shyness compared to social anxiety and whether these are associated with structural changes.

Our aim in this study was therefore to investigate associations between shyness, as opposed to social and trait anxiety, and gray (GM) and white matter (WM) density and functional connectivity using voxel-based morphometry and resting state fMRI respectively in a cohort of healthy young adults. We used Diffeomorphic Anatomical Registration Through Exponentiated Lie algebra (DARTEL) registration, which can achieve more accurate inter-subject registration of brain images [38], and may detect more subtle brain structural changes than those measured using conventional MR images [39]. For functional connectivity analysis we used a seed based strategy [29], [40] whereby regions of interest (ROI) were selected from the structural analysis together with other ROIs previously reported to show activity changes associated with shyness. Overall we hypothesized that shyness would be specifically associated with parallel structural and functional connectivity differences in the limbic and cortical regions of the social brain but that there would be some overlap with social anxiety.

## Methods and Materials

### Participants

A total of 61 healthy volunteers (29 males, 32 females, Mean ± SD = 21.9±1.94 years) were recruited from Sichuan University, Sichuan Normal University, and Southwest Petroleum University, all in Chengdu in China. All participants were healthy, right-handed and of Chinese Han ethnicity, and interviewed with the Structured Clinical Interview by experienced psychiatrists to exclude psychiatric disorders, especially anxiety disorder, according to the Diagnostic and Statistical Manual of Mental Disorder-IV (DSM-IV). All subjects were also free of any neurological problems. The subjects completed self-report measures of shyness (Cheek and Buss Shyness Scale (CBSS, [41]), social anxiety (Liebowitz social anxiety scale (LSAS)) and trait anxiety (Chinese state-trait anxiety inventory (CSTAI-T)). In addition, we used an adapted version of the Eysenck Personality Questionnaire (the edition for adult, EPQ) as an assessment tool for validation of the CBSS [42].

Each participant received a ???40 honorarium for his/her local travel and participation in the MRI component of the study. The study was approved by the local research ethics committee of Sichuan University and written informed consent was obtained from each participant.

### Questionnaire measures

Shyness was measured using the 13 item version of Cheek and Buss Shyness Scale (CBSS) [41], [43], translated into Chinese. The shorter 9 item of this scale has previously been validated in Chinese subjects [44]. This scale is designed to assess both the behavioral and subjective aspects of shyness. Items from this scale include “I find it hard to talk to strangers” and “I feel inhibited in social situations”. Each item was answered on a 0 (extremely uncharacteristic) to 4 (extremely characteristic) scale. In our study scores on CBSS ranged from 13–65 with higher scores reflecting greater shyness. CBSS was developed as a unidimensional measure of shyness, and has been shown to have strong discrimination and convergent validity as well as good internal consistency. Cronbach's alphas in previous studies have typically been 0.78 or higher [45], [46] and 0.91 in Chinese subjects [44].

The Chinese version of Eysenck Personality Questionnaire for Adult (EPQ) was developed by Hunan Medical University in China and contains 88 items self-report measure of characteristics of personality. It is divided into four dimensions as follows: psychosis tendency (psychosis), extroversion or introversion tendency (extroversion), neurosis tendency (neurosis), and the lying tendency of the subjects' responding to the questionnaire (untruthfulness). The subjects were asked to answer question items with “yes” or “no”. The Chinese EPQ has good internal consistency (alpha co-efficient 0.88–0.98) and reliability (0.62–0.86) [42].

The Liebowitz Social Anxiety Scale (LSAS) is comprised of 24-items that assess levels of fear and avoidance in social or performance situations using a 0–3 scale. An overall total score may also be derived by summing the fear and avoidance ratings for all 48 items. The LSAS has been shown to have high internal consistency (alpha coefficient = 0.95; 0.83 and 0.77 for Chinese patients and normal controls), good convergent and discriminant validity, and high test–retest reliability (0.83 for 12-week test–retest) [47]–[49]. A Chinese version of this test has been used in a number of previous studies [50]–[52].

The state-trait anxiety inventory is a frequently used measure of anxiety. The Chinese State-Trait Anxiety Inventory, Trait version, Form Y (CSTAI-T) was designed to measure a stable propensity to experience anxiety, and tendencies to perceive stressful situations as threatening [53]. The scale consists of 20 items, which are scored from 1 to 4, with total possible scores ranging from 20 to 80. Higher scores indicate greater anxiety. This scale has been shown to have high internal consistency (Cronbach's alpha = 0.81; Split-half reliability = 0.83) [54].

### MRI acquisition

Images were acquired on a whole-body 3.0 T MR scanner (Siemens Trio, Erlangen, Germany) with a 12-channel head coil as signal receiver. Foam pads were used to restrict subjects' head motion. High-resolution T1-weighted image was acquired using a magnetization prepared gradient echo sequence (MPRAGE). The sequence parameters were: TR = 1900 ms; TE = 2.26 ms; TI = 900 ms; flip angle = 9°; voxel size = 1×1×1 mm^3^; acquisition matrix = 256×256×176 with a sagittal FOV =  256×256 mm^2^.

MR images detecting BOLD signal were also obtained in the same MRI system with a gradient-echo planar imaging sequence: TR = 2000 ms; TE = 30 ms; FA = 90; acquisition matrix = 64×64; FOV = 240 mm×240 mm; flip angle = 90°; thickness =  5.0 mm; gap = 0 mm; voxel size =  3.75 mm×3.75×5 mm^3^ in-plane resolution. Each brain volume comprised 30 axial slices, and each functional run contained 200 volumes following 5 dummy volumes, with a total scan time of 414 s. All participants were instructed simply to rest with their eyes closed, not to think of anything, and not to fall asleep in particular during the resting-state MR scan.

### Brain morphometry analysis

All MR structural image data were processed using Statistical Parametric Mapping 8 (SPM 8) (http://www.fil.ion.ucl.ac.uk/spm/) running under MATLAB 7.6 (The Mathworks, Natick, MA, USA) to perform VBM. In the preprocessing step of VBM, DARTEL was used to improve inter-subject registration of structural images [38]. The image processing by the SPM 8 software was similar to those used in the studies by Ashburner and Friston [55] and Kosaka et al [56]. Firstly, the artifacts in raw data for each subject were identified and image origin was set at the anterior commissure (AC). Secondly, T1-weighted MR images were first segmented for GM and WM. After segmentation, we generated roughly aligned grey and white matter images of the subjects. Subsequently, structural images of all of subjects (from our shyness brain structural database) were used to make DARTEL templates. The warped data were then smoothed with an 8 mm FWHM, and spatially normalized to Montreal Neurological Institute (MNI) space. In addition, total intracranial volume, total GM volume, and white matter volume across the whole brain were computed from the segmented images for individual participants. To avoid possible edge effects between different tissue types, the absolute threshold masking was used to exclude voxels with GM and WM values of less than 0.1.

Voxel-by-voxel based comparisons of GM density were performed for all of subjects using multiple regression analysis. Firstly, all statistical models included covariates for age, sex and total intracranial volume to account for confounding effects. In order to observe the specific effect of shyness, we then further include the LSAS and CSTAI-T scores as covariates to remove effects of other anxiety. Significance was set at a value of *p*<0.05 (with family wise error corrected at the cluster level).

### Functional connectivity analysis

Functional image preprocessing was carried out using DPARSF (State Key Laboratory of Cognitive Neuroscience and Learning at Beijing Normal University; http://resting-fmri.sourceforge.net/) software. For each participant, the first five images were discarded to ensure steady-state longitudinal magnetization. The fMRI images were then initially corrected for temporal differences and head motion. No translation or rotation parameters in any given data set exceeded ±1.5 mm or ±1.5°. As a result one subject was excluded due to excessive head movement and images from the remaining 60 individuals were included in the subsequent analysis. The following processing steps were used as in our previous studies [57], [58]: slice timing correction; realignment to the middle image; spatial normalization to the MNI echo-planar imaging template; resampling of each voxel to 3×3×3 mm^3^. Lastly, the images were spatially smoothed at 8 mm FWHM.

Functional connectivity was investigated using a temporal correlation approach [59], [60]. Six regions showing GM density changes in our structural analysis (using all of the significantly changed clusters extracted by xjView toolbox) and two regions (bilateral amygdalae, defined by the Automated Anatomical Labeling (AAL) templates implemented in the Wake Forest University (WFU) Pickatlas [61]) reported to show altered activity in shyness by previous neuroimaging studies [20]–[22] were selected as seed regions. Using REST, after bandpass filtering (0.01–0.08 Hz) and linear trend removal, a reference time series for each seed was extracted by averaging the fMRI time series of voxels within each region of interest, as in our previous studies [62], [63]. Six rigid-body head motion parameters, the averaged signals from CSF and WM, and the global brain signal were regressed out using linear regression analysis. Each time series was temporally band-pass filtered (0.01–0.08 Hz). A voxel-wise functional connectivity analysis of the ROIs was used. A correlation analysis was conducted between the seed ROIs and the remaining voxels in the brain. The correlation coefficients were converted to z-values using Fisher's r-to-z transformation to improve normality distribution.

Individual Z value maps in all of participants were gathered using one-sample t-test to identify voxels showing a significant positive or negative correlation with the seed time series (p<0.05, with family-wise error correction for multiple comparisons). Multiple correlation analysis was performed for each voxel on the general linear approach between the shyness scores and functional connectivity. Similar to structural analysis, age, and gender were also modeled as covariates of no interest. Subsequently the LSAS and CSTAI-T scores were also used as covariates to remove effects of anxiety. Significance was set at a value of *p*<0.05 with AlphaSim corrected (combined height threshold of p<0.001 and a minimum cluster size of 24 voxels).

## Results

### Self-report data

Demographic information and CBSS, LSAS and CSTAIT-T scores for the 61 subjects are summarized in Table 1. Scores for the CBSS (65 maximum) covered almost the entire range of 15–65, whereas for LSAS (144 maximum) the range was 1–88 and for CSTAIT-T (80 maximum) the range was 20–64. There were no significant gender differences in age (p = 0.371) or shyness, social anxiety and trait anxiety (CBSS - p = 0.079, LSAS - p = 0.263, CSTAI-T - p = 0.889). Shyness scores from the CBSS were significantly correlated with social anxiety ones from the LSAS (r = 0.376, *p* = 0.003) and also with trait anxiety ones from the CSTAIT-T (r = 0.257, *p* = 0.046). Scores from the LSAS and CSTAIT-T were highly correlated (r = 0.622, *p*<0.001).

**Table 1:** Demographic data for all of the participants.

Subjects	Male	Female	Total	p^a^
Gender (M/F)	29	32	61	-
Age (m ± sd)	22.13±1.90	21.68±1.99	21.90±1.94	0.371
CBSS	40.24±12.64	34.65±11.79	37.31±12.42	0.079
LSAS	37.31±18.89	43.75±25.38	40.68±22.58	0.263
*Total Fear*	17.62±10.75	21.72±13.61	19.77±12.41	0.200
*Total Avoidance*	19.68±11.62	22.00±12.42	20.90±12.01	0.458
CSTAI-T	40.51±11.73	40.90±9.88	40.72±10.71	0.889

a Age and the questionnaire scores were compared using independent sample t-tests.

CBSS = Cheek and Buss Shyness Scale; score ranges from 13 to 65.

LSAS = Liebowitz Social Anxiety Scale; score ranges from 0 to 144. This scale includes two important subscales, namely Total Fear and Total Avoidance, which is derived by summing the fear and avoidance rating for all items.

CSTAI-T = The Chinese State-Trait Anxiety Inventory, Trait version; score ranges from 20 to 80.

### Additional validation of the Chinese version of the CBSS shyness scale

The previous validation of the Chinese version of the CBSS was for the original 9 question version [44] and while our 13 question version showed a similar low significant correlation with social anxiety (r = 0.37 vs. 0.25 in Chou, 2005) we carried out additional correlations with Extroversion and Neuroticism using the EPQ. The correlations found between shyness scores and character traits measured by the EPQ are summarized in Table S1. As expected CBSS scores were negatively correlated with Extraversion (r = –0.745, *p<*0.001) and positively correlated with Neuroticism (r = 0.593, *p*<0.001) consistent with previous research using the English version of the CBSS [63]. Thus overall the 13 question version of the CBSS appears appropriate for measuring shyness in the adult Chinese population.

### Brain morphometry associations with shyness and anxiety

**Figure 1:**
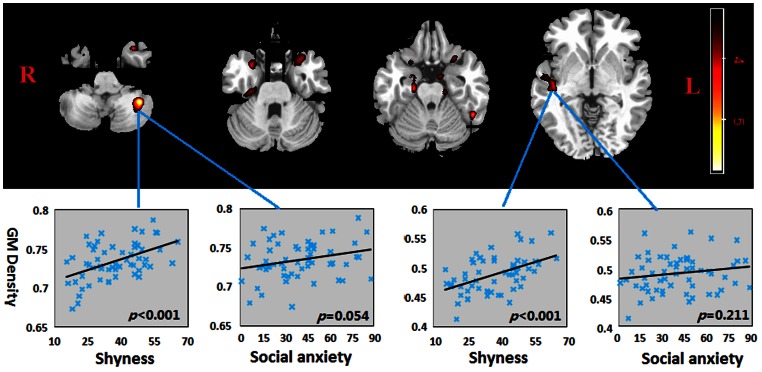
Regions showing positive correlation between GM density and shyness scores without using anxiety scores as covariates. Shyness scores are positively correlated with GM density in the bilateral superior temporal gyri and parahippocampal gyri, and also in the right insula and left cerebellum posterior lobe, when age, gender, and total intracranial volume are used as covariants (at a cluster lever with family wise corrected at p<0.05). Scatter plots show correlations between regional density in left cerebellum posterior lobe and right superior temporal gyrus and shyness and social anxiety scores. Images are presented in radiological orientation. Hot colors indicate brain regions with significant positive correlations with shyness.

Areas where the shyness score was correlated significantly with the GM density are given in Table 2 and Fig. 1. Positive correlations between relative regional density and shyness were seen in the bilateral superior temporal gyri and parahippocampal gyri, and also in the right insula and left cerebellum posterior lobe (at a cluster lever with family wise corrected at *p*<0.05), when controlling for age, sex, and total intracranial volume. No region showed a significant negative correlation between GM density and shyness scores. In order to establish the specificity of shyness compared to anxiety, we additionally regressed out the LSAS and CSTAIT-T scores. In this case significant positive correlations with shyness only remained in the right superior temporal gyrus, and left cerebellum posterior lobe (at a cluster lever with family wise corrected at *p*<0.05). Detailed results are shown in Table 3. The mean regional GM density value in the left cerebellum posterior lobe and right superior temporal gyrus, after correction for age, gender and total intracranial volume, were correlated with shyness, but not with social anxiety scores using Pearson correlation analysis (See Fig. 1).

**Table 2:** Regions showing significant correlations between GM and WM density and Shyness without correction for anxiety scores.

	Correlation type with shyness	Brain Regions	Voxel number	Peak MNI Coordinates			β	P
				x	y	z		
**GM**	Positive	L Cerebellum posterior Lobe	1190	−40	−52	−44	5.80	<0.001
		R Insula	295	43	−23	−2	4.53	<0.001
		R Superior temporal Gyrus (near temporal pole)	842	51	−10	−5	3.60	<0.001
		R Parahippocampal gyrus	1252	22	−4	−23	4.48	<0.001
		L Superior temporal Gyrus (near temporal pole)	1114	−30	15	−28	4.06	<0.001
		L Parahippocampal gyrus	681	−10	−6	−18	3.87	0.014
**WM**	Negative	R Middle temporal WM	349	54	−23	−8	3.99	0.028

Shyness scores are associated with significant positive correlations with regional GM or WM density changes, with age, gender and total intracranial volume as covariates (at a cluster lever FWE corrected at *p*<0.05). L = left and R = right.

**Table 3:** Regions showing significant correlations between GM and WM density and shyness scores with anxiety scores as covariates.

	Correlation type with shyness	Brain Regions	Voxel number	Peak MNI Coordinates			β	P
				x	y	z		
**GM**	Positive	L Cerebellum posterior Lobe	1620	−40	−53	−44	5.26	0.006
		R Superior temporal Gyrus (near temporal pole)	1797	47	−17	−4	4.46	0.003
**WM**	Negative	L Superior temporal WM	722	−48	−57	23	5	<0.001
		L Middle temporal WM	376	−66	−39	−2	4.42	0.019
		R Middle temporal WM	1044	67	−10	−11	4.06	<0.001

Shyness scores are associated with significant positive correlations with regional GM or WM density changes, with age, gender, total intracranial volume, LSAS and CSTAIT-T scores as covariates (at a cluster lever FWE corrected at p<0.05). L = left and R = right.

We also carried out a secondary analysis to establish whether any structural differences were specifically correlated with social anxiety or trait anxiety scores by using regression analysis in SPM 8, with age, gender, shyness and trait or social anxiety scores as covariates. However, there were no brain regions showing significant correlations between GM density and either LSAS or CSTAIT-T scores.

Only the right middle temporal gyrus showed a significant negative correlation between WM density and shyness measures, when we used age, gender, and total intracranial volume as covariates (at a cluster lever with family wise corrected at *p*<0.05; See Table 3). When we further regressed out the LSAS and CSTAIT-T scores, left superior temporal and bilateral middle temporal white matter showed a negative correlation with shyness scores at the same *p*<0.05 level. No regions showed significant associations specifically with LSAS or CSTAIT-T scores.

### Functional connectivity associations with shyness and anxiety

We first established the significant functional connectivity patterns associated with the 8 seed regions chosen (the 6 ROIs showing structural differences associated with shyness and the left and right amygdala). Table S2 and Figure S1 provide details of the resting state connectivity analysis together with the MNI coordinates of the peak foci, and T values. The left cerebellum posterior lobe had significant positive functional connectivity with the right middle frontal gyrus and inferior parietal lobule and left thalamus. It also showed negative functional connectivity with left orbital frontal cortex and right precentral gyrus. The right insula had significant positive functional connectivity with the left cerebellum posterior lobe and negative connectivity with the right posterior cingulate cortex, left superior frontal gyrus and also with part of the left cerebellum. The right superior temporal gyrus showed negative functional connectivity with left superior frontal gyrus and middle temporal gyrus and the right precuneus, while the left superior temporal gyrus showed positive functional connectivity with the right cerebellum posterior lobe, right supramarginal gyrus and left precuneus, and negative functional connectivity with left middle frontal gyrus. The right parahippocampal gyrus had positive connectivity with the left precuneus and negative connectivity with the right superior frontal gyrus, bilateral inferior parietal lobule and left cerebellum posterior lobe whereas the left parahippocampal gyrus showed negative connectivity with the left caudate, precuneus and middle frontal gyrus and right superior frontal gyrus. The right amygdala showed significant negative connectivity with the left precuneus and superior and middle frontal gyri and the left amygdala with the right superior parietal lobule and middle and superior frontal gyri. All regions also showed positive functional connectivity links within themselves.

**Figure 2:**
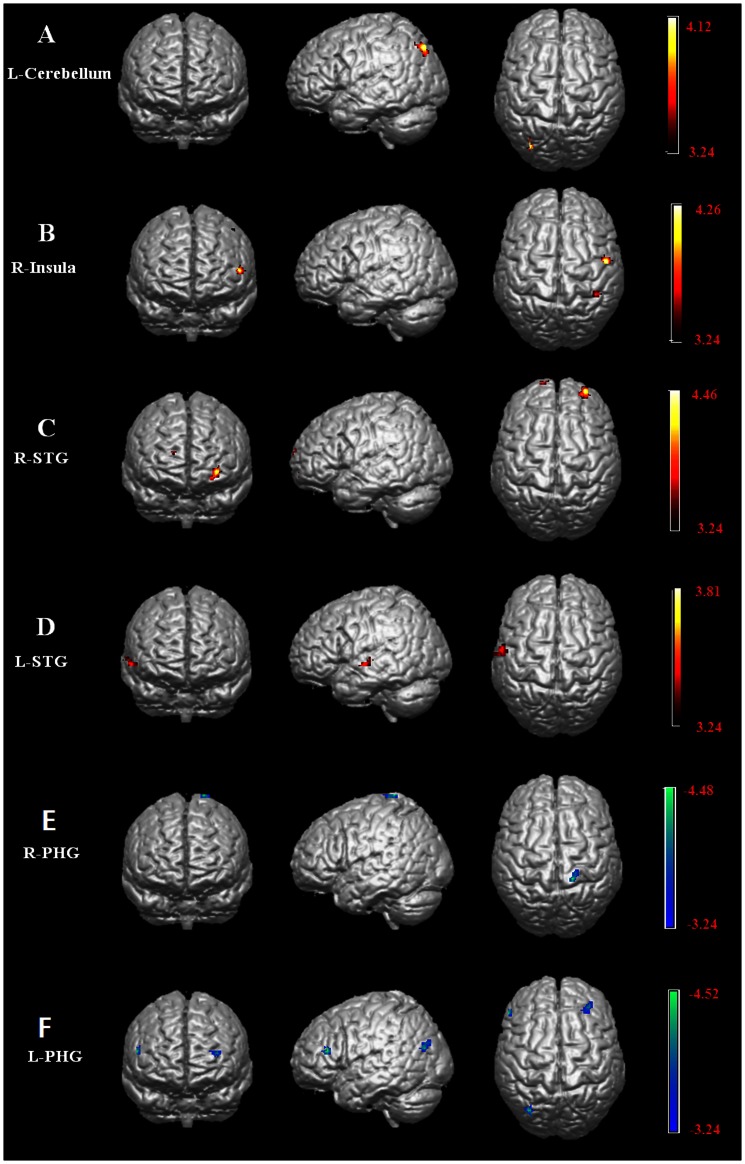
Regions with GM changes showing positive and negative correlations between functional connectivity and shyness without other anxiety correction. Shyness scores are significantly correlated with functional connectivity in left cerebellum (A), right insula (B), bilateral superior temporal gyri (C&D), and bilateral parahippocampal gyri (E & F) seeds. Images are presented in radiological orientation. Hot and cold colors indicate brain regions with significant positive (hot) and negative (cold) correlations with shyness.

**Figure 3:**
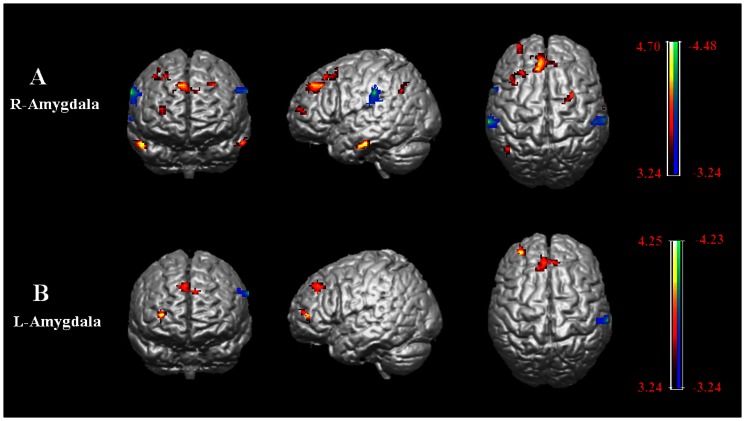
Positive and negative correlations with shyness involving amygdala functional connectivity and without other anxiety correction. Images are presented in radiological orientation. Hot and cold colors indicate brain regions with significant positive (hot) and negative (cold) correlations with shyness using the right (A) and left (B) amygdala as seeds.

**Figure 4:**
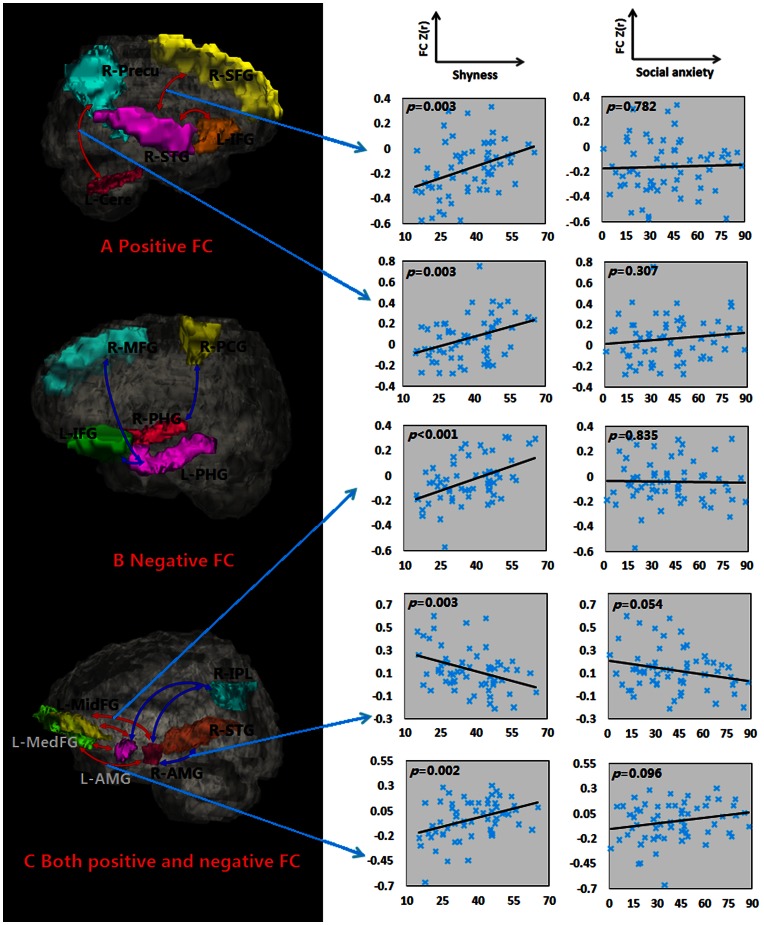
Schematic representation of the functional connectivity associated with shyness. (A) Regions showing positive correlations between functional connectivity and shyness (seeds located in right superior temporal gyrus and left cerebellum posterior lobe; (B) Regions showing negative correlations between functional connectivity and shyness (seeds located in bilaterally parahippocampal gyri); (C) Regions showing both positive and negative correlations between functional connectivity and shyness (seeds located in the bilateral amygdalae). Scatter plots on the right show correlations between regional functional connectivity (FC) value and shyness and social anxiety scores. Abbreviations: L-Cere, left cerebellum posterior lobe; R-Precu, right precuneus; R-STG, right superior temporal gyrus; R-SFG, right superior frontal gyrus; L-IFG, left inferior frontal gyrus; L-PHG, left parahippocampal gyrus; R-PHG, right parahippocampal gyrus; R-PCG, right postcentral gyrus; R-MFG, right middle frontal gyrus; L-AMG, left amygdala; R-AMG, right amygdala; R-IPL, right inferior parietal lobule; L-MedFG, left medial frontal gyrus; L-MidFG, left middle frontal gyrus.

To explore correlates of between shyness scores and functional connectivity, initially a multiple regression analysis with age and gender as covariates was used. Significant positive correlations between shyness scores and functional connectivity were found for four of the seed regions (see Table 4 and Fig. 2): left cerebellum posterior lobe and right precuneus; right insula and left precentral gyrus and inferior parietal lobule; right superior temporal gyrus and left inferior and right superior frontal gyri; left superior temporal gyrus and right middle temporal gyrus. Negative correlations were found for the right parahippocampal gyrus connection with the right postcentral gyrus and left parahippocampal gyrus connection with the left inferior frontal and middle temporal gyri and right middle frontal gyrus (see Fig. 2). For the right amygdala positive correlations were found with connectivity to the left medial and middle frontal gyri, inferior temporal gyrus and inferior parietal lobule and right fusiform gyrus and negative correlations with the right superior temporal gyrus and inferior parietal lobule. For the left amygdala positive correlations were found for connectivity with the left medial and middle frontal gyri and a negative one with the right inferior parietal lobule (see Fig. 3). Figure 4 shows the locations of the different brain seed regions and their functional connections together with their correlations with shyness and social anxiety scores.

**Table 4:** Regions showing significant correlations between functional connectivity and shyness scores without anxiety scores as covariates.

Seed Regions	Connected Location	Correlation type with shyness	Voxel number	Peak MNI Coordinates			β
				x	y	z	
L Cerebellum Posterior Lobe	R Precuneus	Positive	31	33	−78	42	4.12
R Insula	L Precentral gyrus	Positive	31	−46	−9	9	4.26
	L Inferior parietal lobule	Positive	26	−39	−45	45	3.61
L Superior Temporal Gyrus	R Middle temporal gyrus	Positive	38	60	−12	−6	3.81
R Superior Temporal Gyrus	L Inferior frontal gyrus	Positive	52	−30	57	−6	4.46
	R Superior frontal gyrus	Positive	26	15	66	18	3.87
L Parahippocampal gyrus	L Inferior frontal gyrus	Negative	24	−54	33	18	4.52
	L Middle temporal gyrus	Negative	29	−33	−75	18	4.30
	R Middle frontal gyrus	Negative	26	33	36	12	4.10
R Parahippocampal gyrus	R Postcentral gyrus	Negative	26	18	−42	78	4.48
L Amygdala	L Middle frontal gyrus	Positive	31	−33	48	6	4.25
	L Medial frontal gyrus	Positive	77	−6	36	39	3.84
	R Inferior parietal lobule	Negative	47	57	−33	30	4.23
R Amygdala	L Inferior temporal gyrus	Positive	53	−54	−21	−27	4.70
	L Medial frontal gyrus	Positive	120	−3	48	33	4.22
	R Fusiform gyrus	Positive	30	60	−15	−27	4.22
	L Middle frontal gyrus	Positive	78	−30	27	48	3.95
	L Inferior parietal lobule	Positive	28	−42	−63	42	3.57
	R Superior temporal gyrus	Negative	63	39	3	−18	4.21
	R Inferior parietal lobule	Negative	53	66	−27	33	4.04

Shyness scores are associated with significant positive and negative correlations with functional connectivity changes, with age and gender as covariates (In all cases p<0.05 with AlphaSim corrected). L = left and R = right.

To further assess the specificity of correlations with shyness a multiple regression analysis with age, gender, LSAS and trait anxiety as covariates was carried out. Table 5 shows that the positive correlations involving the left cerebellum and right precuneus, the right superior temporal gyrus and superior frontal gyrus and the left amygdala and left middle and medial frontal gyri remained significant. The positive correlation between the right amygdala and left middle and medial frontal gyri, right middle frontal gyrus and left inferior parietal lobule also remained significant as did the negative correlation with the left inferior parietal lobule and right superior temporal gyrus.

**Table 5:** Regions showing significant correlations between functional connectivity and shyness scores with anxiety scores as covariates.

Seed Regions	Connected Location	Correlation type with shyness	Voxel number	Peak MNI Coordinates			β
				x	y	z	
L Cerebellum posterior Lobe	R Precuneus	Positive	28	33	−78	39	4.08
R Superior temporal Gyrus	R Superior frontal gyrus	Positive	33	15	66	18	4.13
L Amygdala	L Middle frontal gyrus	Positive	65	−33	45	3	4.83
	L Medial frontal gyrus	Positive	31	−3	36	42	3.78
R Amygdala	L Middle frontal gyrus	Positive	75	−36	15	51	4.23
	L Inferior parietal lobule	Positive	31	−42	−63	42	4.17
	R Middle frontal gyrus	Positive	30	39	18	51	3.99
	L Medial frontal gyrus	Positive	65	−9	30	39	3.94
	L Inferior parietal lobule	Negative	31	−66	−30	33	3.92
	R Superior temporal gyrus	Negative	24	39	3	−18	3.76

Shyness scores are associated with significant positive and negative correlations with regional functional connectivity changes, with age, gender, LSAS and CSTAIT-T scores as covariates (In all cases p<0.05 with AlphaSim corrected). L = left and R = right.

We also carried out a final analysis to investigate potential correlations between functional connections and social and trait anxiety scores using regression analysis in SPM 8 and with age, gender and shyness and social/trait anxiety scores as covariates. However, no significant correlations were found.

## Discussion

To the best of our knowledge, this is the first study to report structural and functional connectivity differences in the brain associated with shyness. It provides further support for growing evidence that both temperament and personality traits are associated with structural differences [14]–[16] as well as functional connectivity changes [35], [37] in the brain. Furthermore our findings provide strong evidence that in healthy subjects structural and functional connectivity associations with shyness are not also associated with social and trait anxiety scores, lending further support to the view that shyness should be considered as distinct characteristic despite a degree of behavioral correlation with anxiety measures. Overall our findings reveal that shyness, but not social or trait anxiety, is positively correlated with GM density changes in a number of brain regions involved in aspects of social and emotional processing, including the cerebellum posterior lobe and limbic/paralimbic system, namely the superior temporal gyrus, parahippocampal gyrus (PHG), and insula. Furthermore, a number of functional connections involving these regions, and also the amygdala, were either positively or negatively correlated with shyness but not social or trait anxiety scores. These functional connections primarily involved links with medial frontal and parietal regions.

The precise relationship between changes in structural and functional connectivity in the brain and altered responsivity to task-dependent stimuli remains to be fully established. However, it is notable that in our current study shyness was associated with positive GM density and functional connectivity involving a number of brain regions also reported in fMRI studies to be hyper-responsive to social and emotional stimuli in shy individuals [23]. Emotional hyper-reactivity is considered to be characteristic of more pathological forms of social anxiety and is thought to arise from a distorted perception and appraisal of social situations [64]. It has been suggested that shyness is characterized by reduced thresholds for physiological arousal and heightened sensitivity in sensory processing [65]. Consistent with this, our findings of increased GM density and altered functional connectivity in shyness provide additional neuroimaging evidence for the hyper-responsive theory of shyness. The increased GM densities we have found in shy individuals are suggestive of a greater number of neuronal cells/synapses and in general larger populations of neurons can be expected to produce greater outputs than smaller ones [19]. The positive correlation between shyness and functional connectivity in many of these regions with increased GM density also indicates that there are altered spontaneous levels of correlated neuronal activity during resting state. An exception to this is the parahippocampal gyrus where functional connectivity is negatively correlated with shyness Thus in some cases either altered spontaneous activity due to increased GM volume may become less correlated in functionally connected regions, or increased GM volume may sometimes result in reduced activity due to greater numbers of inhibitory cells/synapses. Overall, it seems reasonable to speculate that in most cases increased GM density and functional connectivity in many regions of the social brain in shy individuals could underlie the heightened sensitivity towards social threat stimuli and their resultant social and emotional maladjustment. This potential relationship clearly requires further investigation.

Traditionally, the cerebellum has been considered as a center for motor control and coordination. However, there is increasing neuroanatomical evidence that this region is not only connected with motor pathways but also with other cortical and association areas (including prefrontal cortex, posterior parietal, superior temporal, limbic structures) involved in higher mental functions [66], [67] such as cognitive processing and emotional control [68], [69]. Consistent with these findings, we also found functional connections between left cerebellum posterior lobe and the frontal and parietal cortex and thalamus. It has been proposed that the cerebellum might play a role in anxiety related symptoms, such as hyper arousal, which are present in PTSD and other anxiety disorders [68]. Adult trauma survivors with PTSD have also been reported to have increased blood flow in the cerebellum [70]. Moreover, the cerebellar hyperactivity was reduced by fluoxetine [71]. These findings, together with the results of our investigation, suggest that increased cerebellar GM density and functional connectivity with the precuneus in the parietal cortex may be of psychological significance in shyness.

The increased GM density and functional connectivity in the superior temporal gyrus which is specifically associated with shyness may reflect an enhanced detection of, and sensitivity to social threat. The superior temporal gyrus, is involved in perception of social information, such as eye gaze, body movement and facial expressions [72]. Shyness is also associated with enhanced neural activity in the superior temporal gyrus during processing of faces with happy, fearful and disgust expressions [23]. The superior temporal region has rich projections to the frontal lobes [73], especially the medial prefrontal cortex which influences social reasoning, particularly about one's own, and others', mental states [74]. We found that functional connections between the superior temporal gyrus and the left inferior and superior frontal gyri are negatively coupled suggesting that they are inhibitory. Thus the positive correlation between shyness and this functional connection should lead to reduced activation in the medial frontal cortex and shyness is associated with reduced responses to happy and fearful emotional faces, although not disgust ones, in this region [23]. Thus, overall our findings suggest that shyness may be associated with increased inhibition of medial frontal regions mediating social perception and reasoning by the superior temporal gyrus.

The insula showed a positive correlation between shyness scores and GM density and for its functional connection with the left precentral gyrus. The insula is involved in processing emotional faces, most notably of fear and disgust [75] and in shy subjects the insula has increased activation in response to happy, fearful, angry and disgust face expressions [23]. The insula may also control anxiety through its role in interoceptive processing. It is suggested to generate predictive signaling concerning aversive body states, and this could result in anxiety, negative emotional thoughts and avoidance behavior [75]. Thus increased functional connectivity between the right insula and left precentral cortex may reflect enhanced motor responses evoked by social stimuli such as involuntary mouth or face movements [76].

While the GM density in the parahippocampal gyrus is positively correlated with shyness its functional connectivity with frontal and temporal regions is negatively correlated. The parahippocampal gyrus has extensive efferent and afferent connections with the hippocampus which plays a key role in memory encoding, formation and conditioning [77]. The amygdala-entorhinal pathway and the parahippocampal gyrus are crucially involved in fear conditioning [78] and response inhibition [79]. Increased fearfulness in social situations in shyness may therefore be associated with altered functioning of the parahippocampal gyrus resulting in an enhanced sense of social inadequacy, particularly if exacerbated by negative life experiences [80]. The social inadequacy experienced would then in turn attenuate self-reinforcement and enhance negative self-consciousness in a form a reactive inhibition circle. The parahippocampal gyrus is also involved in the inhibition of inappropriate behaviors, and the prefrontal region in protecting representations of relevant information from interference [79]. Thus reduced functional connectivity between the parahippocampal gyrus and prefrontal cortex associated with shyness may indicate a reduced ability for limiting interference and distractibility and inhibitory control of social stimuli. Overall this might promote increased behavioral inhibition and withdrawal.

The amygdala showed extensive functional connectivity associations with shyness despite a complete absence of structural ones, and enhanced amygdalar responses to novel faces have been reported in many previous studies [20]–[22]. Indeed, the findings by Schwartz et al [22] that enhanced amygdala responses to novel compared to familiar faces occurred in adults categorized as inhibited in early childhood, provided important evidence that shyness can be an enduring and potentially inheritable temperament trait. However, adults with social anxiety disorder also showed heightened amygdalar activation, and this region is well known to be important generally for emotional processing, particularly in association with fear-evoking stimuli [81], [82]. We found that amygdala functional connectivity with the frontal cortex was positively associated with shyness scores while that with the parietal cortex, especially the inferior parietal lobule, was negatively associated. Increased functional connectivity between the amygdala and prefrontal cortex may reflect heightened responses to negative social stimuli and impaired top-down regulation in the brainstem-amygdala-cortical system reported by many studies [83]–[85]. Reduced functional connectivity between the amygdala and parietal cortex is in line with previous findings of activity changes during face perception in social anxiety patients [86] and might be related to impaired emotion recognition and imitation [87]. The absence of structural changes in the amygdala associated with functional connectivity ones in shyness suggests its function may be more easily altered as a function of experience or therapy and is consistent with previous evidence that functional and morphological changes do not always occur in parallel [88].

Although many researchers have attempted to clarify the relationship between shyness and social anxiety by comparing incidence rate and symptomatology [7], [89], the precise relationship between the two remains unclear. In agreement with previous studies we did find a significant, but low correlation between shyness and social anxiety [6], suggesting only limited overlap. Indeed, none of the individuals with shyness scores on the CBSS close to the maximum of 65 scored higher than 88 on the LSAS, which is below the highest social anxiety severity range of 95–144 [90], and none were diagnosed as having social anxiety disorder in accordance with DSM-IV criteria. Thus very shy individuals did not also have serious social anxiety problems. Both shyness and social anxiety disorder show hyperactivity of cortico-limbic circuitry [91], [92], especially in fronto-amygdalar pathway [93], and connectivity between insula and cingulate cortex [94]. Social anxiety disorder and shyness are also associated with increased negative connectivity between the parahippocampal gyrus and the right fronto-parietal network [95]. However, brain morphological changes associated with the two conditions are mainly in the opposite direction, with GM being increased in shyness and decreased in social anxiety disorder [95]–[97]. Thus functional connectivity changes may be contributed to in a different way by GM matter density alterations in shyness and social anxiety. In shyness perhaps increased connectivity is due simply to increased activity resulting from greater numbers of cells/synapses. In anxiety on the other hand reduced numbers of cells and synapses may lead to compensatory changes in synaptic sensitivity. In the case of shyness therefore changes would reflect a natural and non-pathological consequence of having more cells and synapses whereas in anxiety it would reflect attempted compensatory changes resulting from pathology. Thus, despite some striking similarities, it may be the different patterns and causation of GM volume changes which best dissociate shyness from social anxiety.

A limitation of this study is that it only indicates a possible link between structural and functional connectivity in a number of relevant brain which may underlie psychological aspects of shyness since the cross sectional and resting state design cannot establish direct causal roles. Nevertheless, there is a strong overlap between the brain regions and their functional connections that we have identified and those reported by task-dependent studies to have associations with shyness. However, further longitudinal studies using task-dependent approaches will be necessary to establish fully the functional significance of our observations.

In summary, the present study has shown that shyness in adult healthy subjects is associated with structural and functional connectivity changes in a number of brain regions involved in social and emotional processing, including the cerebellum posterior lobe and limbic/paralimbic regions and their connections with the medial frontal and parietal cortices. These changes are only correlated with levels of shyness rather than social or trait anxiety, and may underlie heightened sensitivity towards social threat stimuli as well as help explain why shyness can be an enduring temperament trait.

## Supporting Information

Figure S1 **Resting state functional connectivity maps for the seed regions used.** Seed regions used included the bilateral superior temporal gyri, parahippocampal gyri, right insula and left cerebellum posterior lobe and bilateral amygdalae. Hot and cold colors indicate brain regions with significant positive (hot) and negative (cold) correlations with the selected seed ROI, respectively. Color scales represent T values in each functional connectivity map using one-sample t-tests (*p*<0.05, family wise corrected at criterion for multiple comparisons).
(TIF)

Table S1 **Correlation between shyness and EPQ.** Correlation between EPQ and shyness using the 13 items version of the CBSS in present research are similar with previous research (Schmidt et al., 2008) using the English version of the CBSS.
(DOCX)

Table S2 **Pattern of functional connectivity for seed regions used without other anxiety correction.** Seed regions used areas showing either GM density associations with shyness scores (bilateral superior temporal gyri, parahippocampal gyri, right insula and left cerebellum posterior lobe) or previous evidences for increased activation in shy individuals (bilateral amygdalae). Threshold set at a voxel level with family wise corrected at *p*<0.05. L = left and R = right.
(DOCX)
