# Supplementary figures and images for: Structural and Functional Connectivity Changes in the Brain Associated with Shyness but Not with Social Anxiety
Source: PLoS One. 2013 May 10;8(5):e63151. doi: 10.1371/journal.pone.0063151 (PMC3651210; doi:10.1371/journal.pone.0063151)

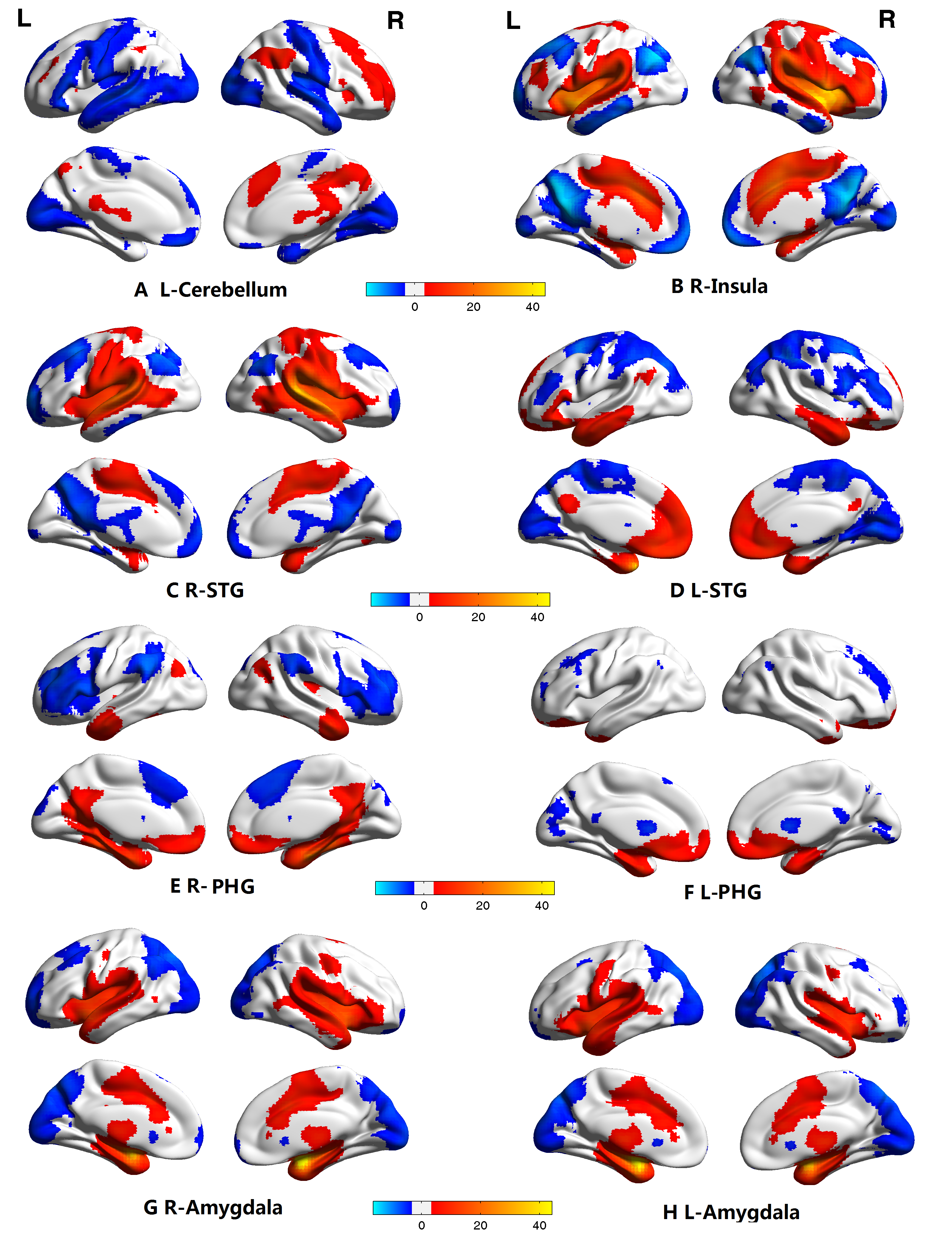

Supplement: Figure S1 — Resting state functional connectivity maps for the seed regions used. Seed regions used included the bilateral superior temporal gyri, parahippocampal gyri, right insula and left cerebellum posterior lobe and bilateral amygdalae. Hot and cold colors indicate brain regions with significant positive (hot) and negative (cold) correlations with the selected seed ROI, respectively. Color scales represent T values in each functional connectivity map using one-sample t-tests (p<0.05, family wise corrected at criterion for multiple comparisons). (TIF) [file pone.0063151.s001.tif]
