# Supplementary material for: Structural and Functional Connectivity Changes in the Brain Associated with Shyness but Not with Social Anxiety
Source: PLoS One. 2013 May 10;8(5):e63151. doi: 10.1371/journal.pone.0063151 (PMC3651210; doi:10.1371/journal.pone.0063151)
Supplement: Table S1 — Correlation between shyness and EPQ. Correlation between EPQ and shyness using the 13 items version of the CBSS in present research are similar with previous research (Schmidt et al., 2008) using the English version of the CBSS. (DOCX) [file pone.0063151.s002.docx]

| **Correlation** | **Extraversion** | **Neuroticism** | **Psychoticism** | **Lie** |
| --- | --- | --- | --- | --- |
| CBSS[The present research] | -.745(*p*＜0.001) | .593(*p*＜0.001) | .191(*p* =.148) | -.181(*p*=.171) |
| CBSS[Schmidt et al., 2008] | -.608(*p*＜0.01) | .474(*p*＜0.01) | -.212(*p*＜0.01) | -.010 |
