# Supplementary material for: Structural and Functional Connectivity Changes in the Brain Associated with Shyness but Not with Social Anxiety
Source: PLoS One. 2013 May 10;8(5):e63151. doi: 10.1371/journal.pone.0063151 (PMC3651210; doi:10.1371/journal.pone.0063151)
Supplement: Table S2 — Pattern of functional connectivity for seed regions used without other anxiety correction. Seed regions used areas showing either GM density associations with shyness scores (bilateral superior temporal gyri, parahippocampal gyri, right insula and left cerebellum posterior lobe) or previous evidences for increased activation in shy individuals (bilateral amygdalae). Threshold set at a voxel level with family wise corrected at p<0.05. L = left and R = right. (DOCX) [file pone.0063151.s003.docx]

| **Seed Regions** | **Connection type with shyness** | **Connected Location** | **Voxel number** | **Peak MNI Coordinates** | | | **T** | **p** |
| --- | --- | --- | --- | --- | --- | --- | --- | --- |
|  |  |  |  | **x** | **y** | **z** |  |  |
| L Cerebellum posterior Lobe | Positive | L Cerebellum posterior lobe | 7912 | -39 | -54 | -45 | 53.70 | <0.001 |
|  |  | R Middle frontal gyrus | 2689 | 39 | 42 | 42 | 10.55 | <0.001 |
|  |  | R Inferior parietal lobule | 1047 | 54 | -45 | 39 | 9.39 | <0.001 |
|  |  | L Thalamus | 430 | -21 | 6 | 21 | 5.85 | 0.003 |
|  | Negative | R Precentral gyrus | 13238 | 48 | -18 | 63 | 8.69 | <0.001 |
|  |  | L Orbital frontal cortex | 492 | -6 | 45 | -24 | 7.11 | <0.001 |
| R Insula | Positive | R Insula | 19249 | 42 | 4 | -3 | 44.23 | <0.001 |
|  |  | L Cerebellum posterior lobe | 161 | -21 | -66 | 51 | 5.63 | 0.005 |
|  | Negative | R Posterior cingulate | 11946 | 3 | -57 | 12 | 16.42 | <0.001 |
|  |  | L Cerebellum posterior lobe | 851 | -3 | -57 | -51 | 13.22 | <0.001 |
|  |  | L Superior frontal gyrus | 5094 | 21 | 42 | 48 | 12.85 | <0.001 |
| L Superior temporal gyrus | Positive | L Superior temporal gyrus | 10798 | -30 | 15 | -30 | 50.04 | <0.001 |
|  |  | R Cerebellum posterior lobe | 241 | 30 | -87 | -36 | 7.76 | <0.001 |
|  |  | R Supramarginal gyrus | 251 | -60 | -57 | 30 | 6.74 | <0.001 |
|  |  | L Precuneus | 277 | -6 | -51 | 30 | 5.64 | 0.012 |
|  | Negative | L Middle frontal gyrus | 15047 | 30 | 6 | 57 | 11.22 | <0.001 |
| R Superior temporal gyrus | Positive | R Superior temporal gyrus | 16179 | 60 | -30 | 12 | 35.64 | <0.001 |
|  | Negative | R Precuneus | 16784 | 33 | -72 | 42 | 12.48 | <0.001 |
|  |  | L Superior frontal gyrus | 2035 | -21 | 66 | 6 | 11.36 | <0.001 |
|  |  | L Middle temporal gyrus | 241 | -57 | -33 | -18 | 6.60 | <0.001 |
| L Parahippocampal gyrus | Positive | L Parahippocampal gyrus | 6339 | -12 | -6 | -21 | 48.96 | <0.001 |
|  | Negative | L Caudate | 117 | 0 | 6 | 9 | 6.55 | 0.001 |
|  |  | R Superior frontal gyrus | 782 | 24 | 39 | 33 | 5.26 | 0.039 |
|  |  | L Precuneus | 1696 | -3 | -73 | 39 | 5.13 | 0.042 |
|  |  | L Middle frontal gyrus | 430 | -27 | 27 | 30 | 5.18 | 0.049 |
| R Parahippocampal gyrus | Positive | R Parahippocampal gyrus | 11058 | 24 | -21 | -27 | 34.52 | <0.001 |
|  |  | L Precuneus | 199 | -39 | -75 | 36 | 6.05 | 0.003 |
|  | Negative | R Superior frontal gyrus | 7562 | 36 | 51 | 18 | 9.37 | <0.001 |
|  |  | L Inferior parietal lobule | 880 | -60 | -42 | 33 | 9.12 | <0.001 |
|  |  | L Cerebellum posterior lobe | 1033 | -24 | -75 | -42 | 7.13 | <0.001 |
|  |  | R Inferior parietal lobule | 633 | 57 | -39 | 39 | 7.07 | <0.001 |
| L Amygdala | Positive | L Amygdala | 15931 | -24 | -3 | -18 | 54.81 | <0.001 |
|  | Negative | R Superior parietal lobule | 10911 | 21 | -66 | 60 | 10.69 | <0.001 |
|  |  | R Middle frontal gyrus | 431 | 30 | 3 | 63 | 5.88 | 0.006 |
|  |  | R Superior frontal gyrus | 449 | 15 | 54 | -24 | 5.76 | 0.008 |
| R Amygdala | Positive | R Amygdala | 16776 | 27 | 0 | -18 | 61.21 | <0.001 |
|  | Negative | L Precuneus | 10058 | 0 | -75 | 54 | 10.95 | <0.001 |
|  |  | L Superior frontal gyrus | 1422 | -24 | 60 | 5 | 6.65 | <0.001 |
|  |  | L Middle frontal gyrus | 738 | -30 | 12 | 51 | 5.86 | 0.006 |
